# Novel conductive polycarbazolic polymer embedded with palladium nanoparticles as a highly sensitive electrochemical sensor for hydrazine detection

**DOI:** 10.1038/s41598-025-25980-8

**Published:** 2025-11-26

**Authors:** Moghadaseh Aghaei Araei, Moslem Mansour Lakouraj, Shahram Ghasemi, Rahman Hosseinzadeh

**Affiliations:** 1 Laboratory of polymer chemistry, Department of Organic Chemistry, Faculty of Chemistry, University of Mazandaran, Babolsar, Iran; 2 Department of Nanochemistry, Faculty of Chemistry, University of Mazandaran, Babolsar, Iran

**Keywords:** Carbazole-based electrode, Electrochemical sensor, Pd nanoparticles, Hydrazine detection, Electrocatalysis, Environmental monitoring, Chemistry, Materials science, Nanoscience and technology

## Abstract

To meet the demand for sensitive, trace-level hydrazine detection, we present a novel, high-sensitivity electrochemical sensor. We synthesized a novel carbazole-based conducting polymer (CRCP) as a distinctive host matrix, demonstrating exceptional stability, high redox potential, and excellent electrochemical properties. Subsequently, we developed an innovative electrochemical sensor by controllably embedding Pd nanoparticles (Pd) into this as-synthesized CRCP matrix using a simple cyclic voltammetry (CV) method to create a highly efficient Pd/PCz@GCE system for sensitive detection of hydrazine in alkaline media. The modified electrode exhibited significant electrocatalytic activity towards hydrazine oxidation. Notably, the FE-SEM analysis revealed an even distribution of spherical Pd nanoparticles (measuring 62–98 nm) across the polymer surface, a key factor that enhances electrocatalytic performance. It has a low detection limit of 0.084 μM, high sensitivity (56.64 μA Μm^−1^ cm^−2^), and excellent selectivity and is comparable or superior to previously reported sensors. The sensor offers a broad linear detection range (0.3–100 μM), and its performance is reproducible, repeatable, and stable. Kinetic studies revealed a diffusion-controlled process at the electrode surface. The effectiveness of the Pd/PCz@GCE was evaluated in real samples. These results indicate that the Pd/PCz@GCE sensor could serve as a reliable and practical platform for sensitive hydrazine detection in environmental analysis.

**Supplementary Information:**

The online version contains supplementary material available at 10.1038/s41598-025-25980-8.

## Introduction

In recent decades, environmental pollution has been a major global challenge, affecting biological diversity, the environment, and human health^1,2^. Common chemical contaminants, such as heavy metal ions, biocides, organic pollutants, and hydrazine, pose significant risks to human health and the environment. This is because of their toxic and carcinogenic effects^3,4^. Among them, hydrazine is a liquid chemical with widespread industrial uses, but it is highly toxic and carcinogenic. Exposure to hydrazine can cause poisoning, respiratory problems, and damage to the liver, lungs, and kidneys. Therefore, a reliable method for detecting hydrazine in the environment is crucial for human health protection. Several methods have been introduced for the determination of hydrazine, such as flow injection, chromatography, chemiluminescence, Raman spectroscopy, fluorescence, and electrochemical analyses^5–8^. Electrochemical detection is a popular choice because of its advantages, such as low cost, in situ analysis, sensitivity, and appropriate response time^9–11^. Additionally, the electrooxidation of hydrazine produces environmentally friendly compounds such as water and nitrogen^12^. However, hydrazine oxidation on electrode surfaces, especially on a GCE, causes a high overpotential. To overcome this issue, modifying electrodes with suitable compounds such as conductive polymers (CPs) and metal nanoparticles (MNPs) is a viable approach^3,13–20^. The combination of nanosized materials (NMs) and CPs has been applied to improve the performance of pollutant-detecting sensors, including electrochemical sensors^14,21–23^. In fact, CPs, with their highly accessible surface area, low ohmic drop, and chemical stability, are suitable host matrices for immobilizing MNPs^24^. The catalytic activity of MNPs in electrodes modified with composites depends on the shape and size of the particles and the nature of the CP.

The use of CPs as stabilizers can prevent MNP aggregation, which affects their specific activity and performance in electrochemical sensors^25,26^. Compared with other CPs, CRCPs have gained attention because of their stability and higher redox potential. Indeed, CRCPs exhibit good electrochemical properties due to their high positive hole transport mobility^27,28^. Among the nanosized metals used in electrochemical sensors, noble metals such as Pd, Ag, Pt, and Au are of particular interest because of their high reduction potential, low toxicity, and size-dependent electrical properties^26,29^. Palladium nanoparticles (Pd NPs) are known for their high efficiency in catalyzing the electrochemical oxidation of hydrazine^30–35^. To date, a few researchers have reported the use of conducting polymers with embedded Pd NPs to increase the efficacy of electrodes for voltammetric determination of hydrazine^36,37^.

Recent advances in electrochemical hydrazine sensing have improved performance through the deliberate design of engineered nanostructures; however, each reported approach still shows inherent limitations and trade-offs. Ag@ZIF-8/SPE sensors offer low detection limits but operate over narrow ranges, with limited validation on real samples^38^. ZnFe_2_O_4_/RGO composites improve sensitivity but are still limited to sub-millimolar detection levels^39^, while MIP‑based Co–Ba–SnO_3_/f‑MWCNT designs offer high selectivity at the expense of complex, multi-step manufacturing^40^. Ultra-sensitive AgNPs@PEI‑HNT composites attain record sensitivities but depend on costly silver nanostructures and labor-intensive production^41^. ZnO–Cu/GCE electrodes perform reliably in real samples but have high detection limits and narrow measurement ranges^42^. To address these ongoing challenges, we introduce a Pd/PCz@GCE platform in which an N_‑_substituted polycarbazolic polymer creates a durable, highly conductive matrix that evenly hosts Pd nanoparticles. This design combines low-cost, scalable manufacturing with ultra-low detection limits, a broad linear detection range, and long-term stability in real sample analysis—features that, to our knowledge, have not been previously integrated in hydrazine sensors.

In this study, we present a novel and highly efficient electrochemical sensor for hydrazine detection, addressing the critical need for sensitive and reliable monitoring of this hazardous pollutant. Our approach involves modifying a glassy carbon electrode with a newly developed N-substituted polycarbazolic conductive polymer. This unique polymer matrix is distinguished by its exceptional properties, including a high surface area, low ohmic drop, robust aromatic structure, and superior thermal stability, which together create an ideal platform for enhanced electron transfer and immobilization. Importantly, we incorporate palladium nanoparticles (Pd NPs) into this polymer matrix. While Pd NPs are recognized for their catalytic activity, their synergistic combination with our particular polycarbazolic polymer marks a notable advancement over current methods. This innovative composite design effectively overcomes limitations commonly faced by traditional hydrazine sensors, such as limited sensitivity, poor selectivity, or stability problems. The resulting Pd/PCz@GCE sensor exhibits significantly enhanced sensitivity, selectivity, and durable detection capabilities for hydrazine at micromolar levels, offering a practical and superior solution for environmental analysis.

## Results and discussion

### Characterization

#### Synthesis and characterization of *N*-(4-nitrophenyl)carbazole and *N*-(4 aminophenyl)carbazole

*N*-(4-nitrophenyl)carbazole was synthesized via the reaction of carbazole as a nucleophile and 4-fluoronitrobenzene as an aromatic electrophilic substrate under alkaline conditions. *N*-(4-Aminophenyl)carbazole was then synthesized by further hydrogenation of the precursor carbazole with hydrazine as the hydrogen source and Pd/C as the catalyst. The melting point of *N*-(4-aminophenyl)carbazole (T_m_ = 106–107 °C) and FT-IR spectra (3421, 3356 cm^−1^; NH_2_ stretch) revealed the successful synthesis of *N*-(4-aminophenyl)carbazole, which was consistent with previous studies^43,44^.

#### Synthesis and characterization of the polymer

The procedure for the polymerization of the carbazolic monomer leads to different possibilities for the binding of radical species, which are produced as the result of the mechanism that starts with APS as the initiator^27^. The carbazolic monomer has three possible sites that may carry the unlocalized electron of APS, resulting in the formation of radicals. The amine part of the aminophenyl and the C3 and C6 of the carbazole rings can be easier joints than the other sites^45^. These sites consider the structure, including the bis carbazole and polyaniline-like moieties, of the polymer (Fig. S1).

As the first revealed characteristic, the solubility of the resulting polymer in DMSO indicates its low degree of polymerization, with minimal π–π stacking of long chains, which in turn enables the polymer to dissolve easily.

Fig. S2A shows the FT-IR spectra of the poly**(**N-(4-aminophenyl)carbazole) polymer. The broad peak at approximately 3444 cm^−1^ corresponds to the N–H stretching vibration of the secondary amine groups in the polymer. The peaks at 1598 and 1507 cm^−1^ are assigned to the C=N and C=C stretching vibrations of the quinoid and benzenoid rings, respectively. The peak at 1224 cm^−1^ is assigned to C–N stretching vibrations, and the peak at 847 cm^−1^ can be attributed to the out-of-plane bending vibration of the benzene ring. Additionally, the characteristic peak at approximately 1110 cm^−1^ indicates an electron-like band, which is considered to be a measure of delocalization^27,45,46^.

The XRD pattern of the carbazole-based polymer is also shown in Fig. S2B. Four broad peaks are observed at approximately 2θ = 12, 20, 45, and 72° which indicate the amorphous and semicrystalline nature of the polymer structure. The intense strong peak at position 20 and other weak peaks are the reflection of parallel planes of the polymer chain and the periodicity perpendicular to the polymer chain, respectively^47^.

The average crystallite size of the synthesized polymer was calculated via the Debye–Scherrer equation (Eq. (1))^48^:

1 $${\text{D}} = \, \left( {{\text{k}}\lambda /\beta {\text{ cos }}\theta } \right)$$

where λ is the X-ray wavelength (1.54178 Å), K is the Scherrer constant, which is dependent on the crystallite shape (K = 0.94), β is the full width at half maximum, and θ is the Bragg angle at the center of the corresponding peak. The average size of the crystallites (D) obtained with Eq. (1) for the carbazolic polymer was 44.30 nm. The surface morphology of the polymer was examined via field-emission scanning electron microscopy, and the images are presented in Fig. S2C. The FE-SEM images revealed flake-like self-assembled nanoparticles that formed irregular structures. The thermal stability and characteristics of the polymer were also analyzed, and the TGA/DSC thermograms are shown in Fig. S3A. According to the DSC thermogram, the polymer has two endothermic structural changes without any noticeable weight loss at temperatures below 250 °C, indicating physical attributes such as softening and melting points. The TGA thermogram of the polymer revealed an approximately 27% weight decrease in the endothermic degradation step in the 295–405 °C temperature range and a nearly 30% further weight loss corresponding to another degradation step, which occurred in the analyzed temperature range.

Figure S3B shows the UV‒Vis spectra of the carbazole monomer and the synthesized polymer. The monomer and polymer were separately dissolved in DMSO, filtered to obtain a clear solution, and then subjected to UV‒Vis spectroscopy at room temperature. The monomer and polymer show characteristic bands with maximum peaks at 260 and 266 nm, respectively, which can be attributed to π → π^*^ and a polaron → π^*^ conduction band transitions^49^. The broadened absorption bands at 290 nm for the monomer and 295 nm for the polymer correspond to the valance band-to-conduction band transition. In addition, weak broad peaks in the 325–375 nm range for both compounds can be assigned to the polaron-to-π^*^ conduction band and excitonic transitions^45,49^. The bathochromic and hyperchromic shifts in the maximum absorption band of the polymer compared with that of the monomer reveal the polymerization of the monomer and the extension of the π-conjugation length^45^.

#### Characterization of modified electrochemical electrodes

**Fig. 1:**
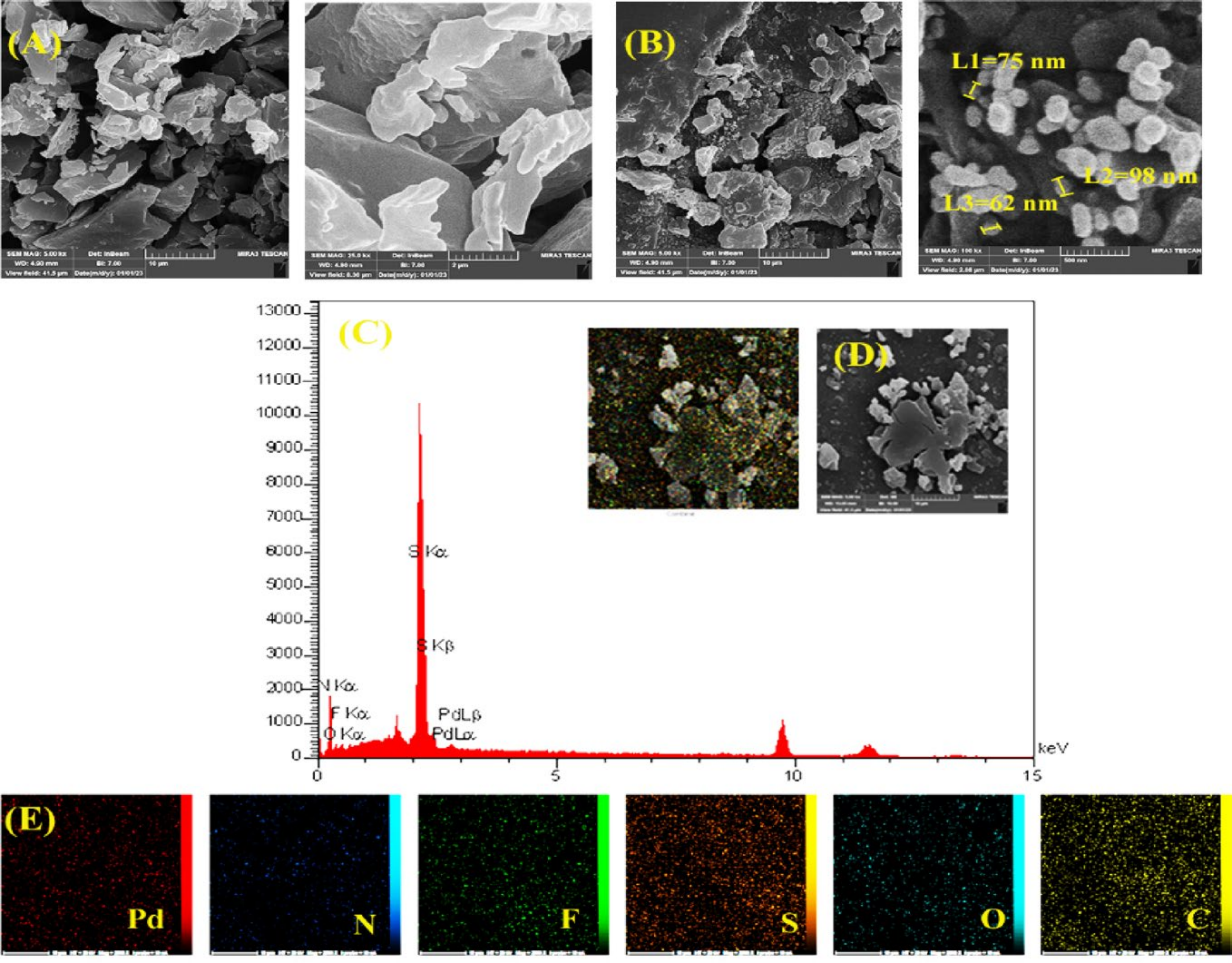
FE‒SEM images of (**A**) PCz@GCE and (**B**) Pd/PCz@GCE, (**C**) EDX spectrum, (**D**) total elemental mapping and (**E**) detailed elemental mapping of Pd/PCz@GCE.

The formation of carbazolic polymers on the surface of the GCE and the Pd promotion of the polymer-modified electrode were reproducibly achieved via CV. To describe the morphology, size, and distribution of the electrodeposited Pd nanoparticles, detailed Field Emission Scanning Electron Microscopy (FE-SEM) was conducted) Fig. 1B. The electrochemically adsorbed polymer was well distributed on the surface of the GCE, resulting in micron- and submicron-scale polymer particles. After modification of the PCz@GCE with Pd nanoparticles, a widespread distribution of spherical particles was observed at the electrode surface.

The FE-SEM images of Pd/PCz@GCE (Fig. 1B) clearly show that the electrodeposited palladium nanoparticles mainly have a spherical or nearly spherical shape. An estimated size from the FE-SEM images indicates that these Pd nanoparticles typically range from 62 to 98 nm in diameter. More importantly, these images display a highly uniform and extensive distribution of nanoparticles over the entire surface of the PCz@GCE, pointing to excellent dispersion and little aggregation. Elemental mapping analysis confirms uniform distribution of Pd across the modified electrode surface (Fig. 1E). Such a consistent and even dispersion of catalytically active Pd nanoparticles is essential for maximizing the active surface area and achieving efficient electrocatalytic performance for hydrazine detection.

Figure 1C, D and E show the EDS spectrum of Pd/PCz@GCE with its composition, total elemental mapping, and elemental map in detail, respectively. The EDS spectrum of Pd/PCz@GCE indicates the presence of Pd with an atomic percentage of 0.66. The high percentages of S and F are due to the synthesis procedure in which the TBABF4 in DMSO was used as a supporting electrolyte for the electrodeposition of the polymer on the electrode surface. The mapping of the electrode surface shows the distribution and abundance of elements via the characteristic X-ray emissions.

### Electrochemical evaluations

#### Electrochemical behavior of the modified electrode

**Fig. 2:**
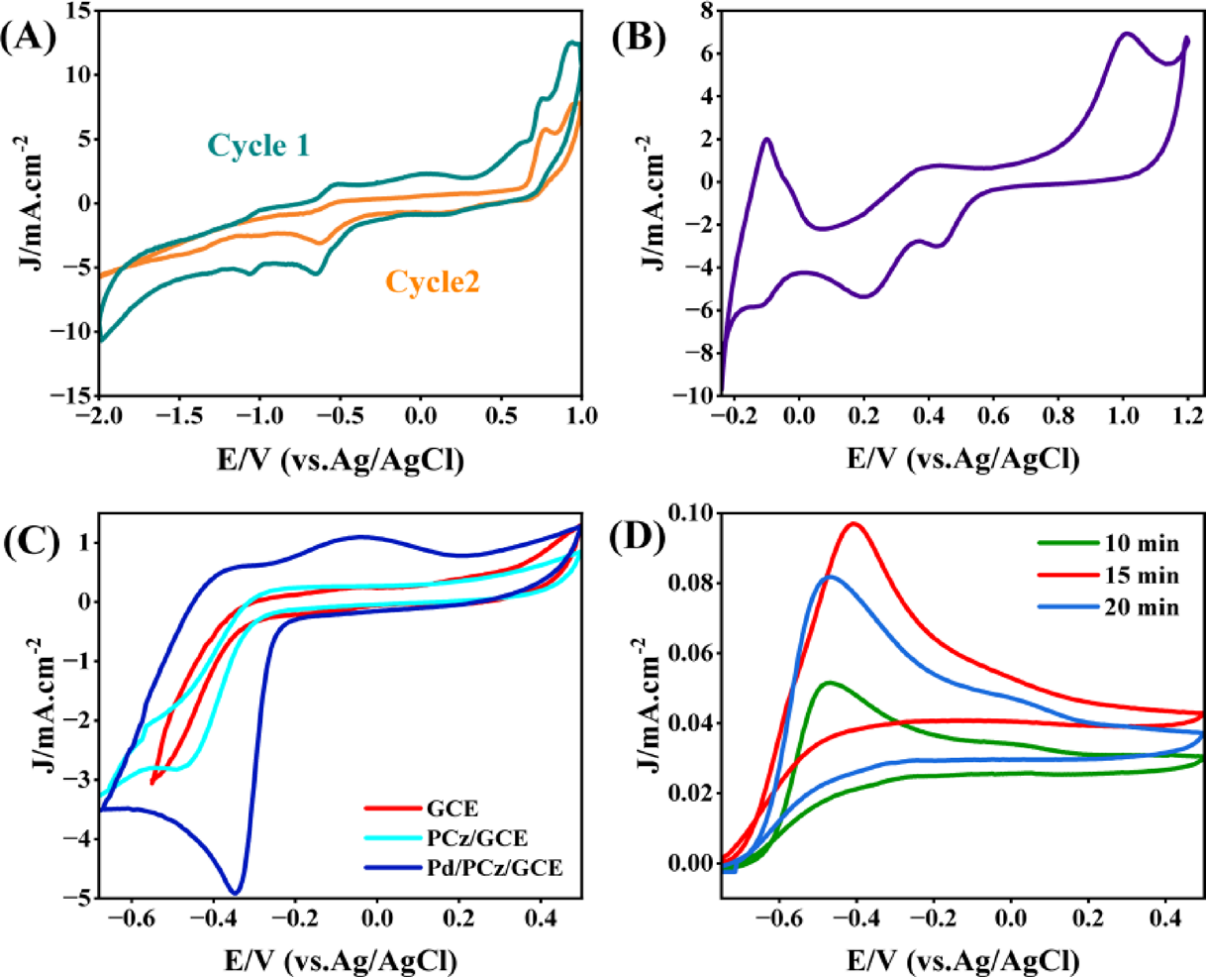
(**A**) First two voltammograms of carbazolic polymer deposition in 1 mM TBABF_4_/DMSO solution at 50 mV s^−1^ ,Cycle 1 and Cycle 2, (**B**) CV curves of Pd nanoparticle electrodeposition on the surface of PCz@GCE, (**C**) voltammograms of different electrodes in 0.1 M NaOH solution, and (**D**) voltammograms of Pd/PCz@GCE prepared at different immersion times (in 5 mM PdCl_2_ acidic solution at 15 min, 10 min, and 20 min) in 0.1 M NaOH solution with 1 mM hydrazine concentrations at a scan rate of 50 mV s^−1^.

Figure 2A shows the first two consecutive CV curves of the carbazolic polymer in a 1 mM TBABF_4_/DMSO solution at a potential scan rate of 50 mV s^−1^.

Two oxidation peaks at − 0.55 and 1 V are observed, which can be attributed to oxidative NH_2_−carbazole and biscarbazole jointing reactions while CV is carried out. The electrodeposition process results in the formation of a more complex and extended polymer on the surface of the GCE, which is based on bis-carbazole and polyaniline-like moieties (Fig. S7).

The cyclic voltammogram for electrodeposition of Pd nanoparticles on the surface of PCz@GCE is shown in Fig. 2B. There are three current features characteristic of the reduction of Pd(II), oxidation of Pd and adsorption‒desorption of hydrogen at 0.43, 1 and -0.11 V, respectively. Applying a potential from high to low values leads to the reductive deposition of Pd species on the surface of the polymer-modified electrode. At a negative potential, the reduction of H^+^ ions takes place on the surface of Pd, while when the potential is applied in the opposite direction, the peak of hydrogen oxidation appears, and then Pd oxidation occurs. Repeating the potential cycle in the first applied direction reduces the former oxidized Pd to Pd^0^, followed by reductive adsorption of hydrogen. Broad redox couples at 0.1 to 0.6 V verify the presence of electrochemically synthesized carbazolic polymers on the GCE.

Figure 2C shows the cyclic voltammograms of the GCE, PCz@GCE and Pd/PCz@GCE in basic media (0.1 M NaOH) at 50 mV s^−1^. For the GCE and PCz@GCE, no peaks are observed, whereas the redox peaks of Pd in Pd/PCz@GCE are clear.

To prepare the Pd/PCz@GCE electrode, the PCz@GCE electrode was immersed in a 5 mM PdCl_2_ solution for different durations (10 min, 15 min, and 20 min). After the reduction of the palladium on the surface of the electrodes, their CV curves were recorded in 0.1 M NaOH solution with 1 mM hydrazine. The highest current density was observed for the Pd/PCz@GCE electrode immersed for 15 min. Therefore, the optimal time for Pd deposition was 15 min (Fig. 2D). The amount of immobilized Pd nanoparticles is a key parameter in detecting hydrazine. Properly optimizing this parameter can significantly enhance the sensor’s performance in terms of sensitivity, selectivity, and detection limit, as loading too low may result in insufficient catalytic activity, leading to poor sensitivity. In contrast, excessive loading may cause catalyst aggregation and hinder the catalytic reaction due to a reduced contact area or reduced active surface area^50^.

**Fig. 3:**
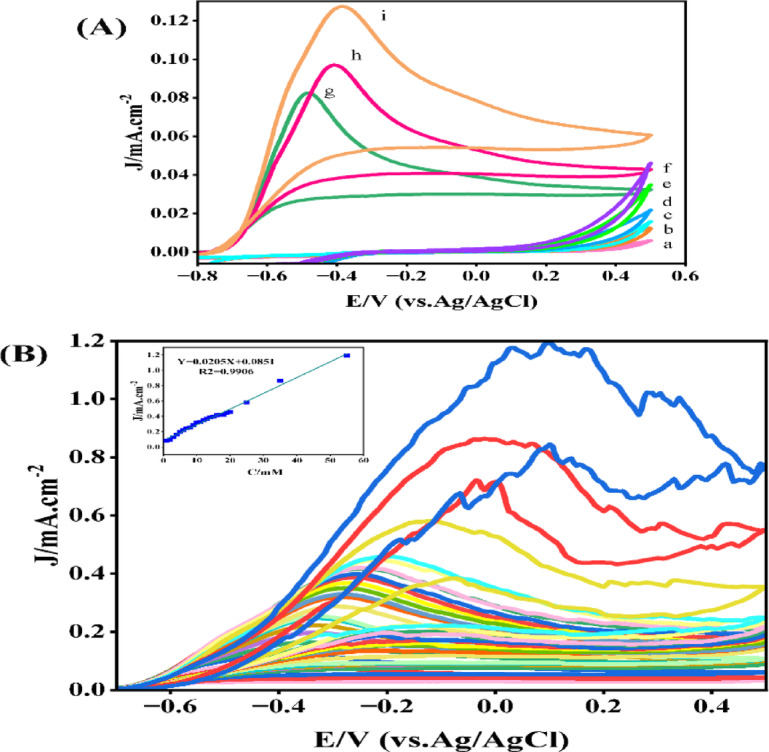
(**A**) Voltammograms of the GCE (a-c), PCz@GCE (d-f), and Pd/PCz@GCE (g-i) at different hydrazine concentrations: 0.25 mM, 1 mM, and 2 mM. (**B**) CV curves of Pd/PCz@GCE at various concentrations (1–55 mM) of hydrazine (inset: calibration plot).

The electrochemical behaviors of the GCE, PCz@GCE, and Pd/PCz@GCE at different hydrazine concentrations (0.25 mM, 1 mM, and 2 mM) in 0.1 M NaOH solution are shown in Fig. 3A. CGE (curves a-c) and PCz@GCE (curves d-f) clearly have no effect on the electrochemical oxidation of hydrazine in the applied potential range, but in the case of Pd/PCz@GCE, the anodic peaks are observed in the corresponding voltammograms (curves g-i). This significant change can be attributed to the presence of Pd nanoparticles embedded and distributed in the polycarbazole matrix, which facilitates the oxidation of hydrazine.

#### Hydrazine concentration effect

The cyclic voltammograms of Pd/PCz@GCE in the presence of hydrazine at various concentrations are depicted in Fig. 3B. As the concentration of hydrazine increases, the current density of the oxidation peaks increases, and simultaneously, the anodic peaks shift to higher potentials. The shift of the anodic peaks to higher potentials with rising hydrazine concentration can be attributed to the fact that higher concentrations of hydrazine promote a faster overall reaction rate at the electrode surface. This acceleration in reaction kinetics requires a more positive potential to drive the reaction forward, as reflected in the shift of the anodic peaks to higher potentials. Specifically, this behaviour is characteristic of irreversible or quasi-reversible electrochemical processes where the kinetic of the oxidation reaction is limiting factor; an increased potential is necessary to overcome the higher kinetic barriers, resulting in a positive shift in the peak potential. This kinetic effect is a well-documented phenomenon, often observed when charge transfer rates depend on concentration at higher concentrations^51^. The inset chart in Fig. 3B reveals a good linear relationship between the current density of the oxidation peaks and the hydrazine concentration over a wide range (up to 55 mM). Although a strong linear correlation is observed, the slope of the calibration curve varies across different concentration ranges. At lower hydrazine levels, electrocatalytic oxidation mainly depends on reaction kinetic, resulting in an efficient process and a steeper slope in the calibration curve. As the hydrazine concentration increases, particularly at higher levels, intensified oxidation results in the rapid and substantial production of nitrogen gas (N_2_) at the electrode surface which temporarily blocks accessible active sites and limit mass transfer for hydrazine. This limitation not only reduces the slope of the calibration curve at higher concentrations, but also introduces oscillations and noise in the cyclic voltammograms, as gas bubbles intermittently interfere with electron transfer^52,53^.

#### Scan rate effect

**Fig. 4:**
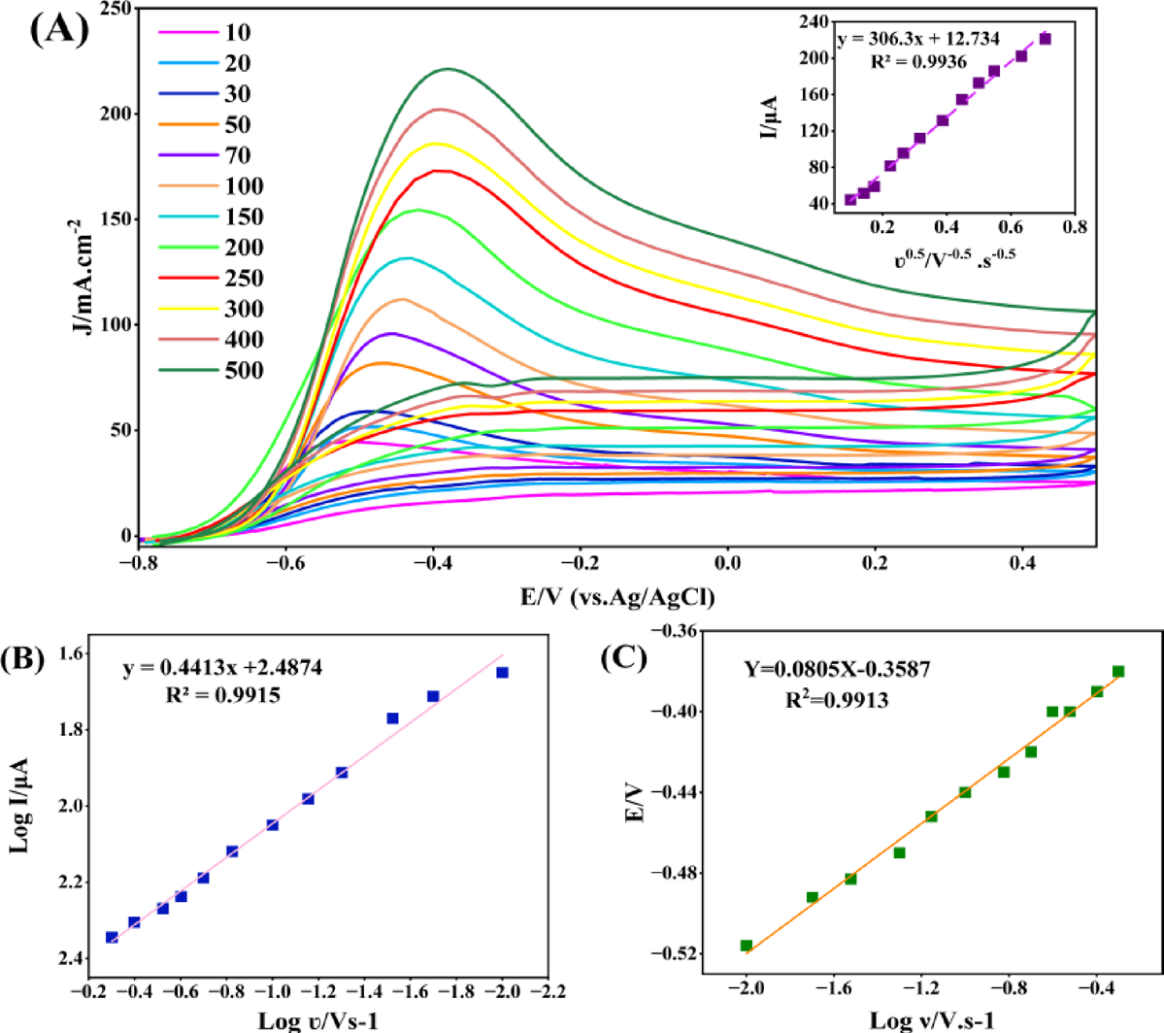
(**A**) CV curves for the oxidation of hydrazine (1 mM) on Pd/PCz@GCE in 0.1 M NaOH solution in the range of 0.01–0.5 V s^−1^ and the relationship between ʋ^1/2^ and I_p_ (inset chart). Plots of (**B**) Log I and (**C**) E vs. Log ʋ.

To investigate the dependence of the current density and peak position for hydrazine oxidation on the scan rate, voltammograms of Pd/PCz@GCE were recorded in the presence of 1 mM hydrazine in a 0.1 M NaOH solution in the range from 0.01 to 0.5 V s^−1^ (Fig. 4A). For the anodic peak, higher current densities are observed with increasing potential scan rate, and the potentials tend to shift to higher values as a result of limitations in the reaction kinetics. The relationship between the current density of the oxidation peak (I_p_) and the scan rate square root (ʋ^1/2^) reveals the controlling mechanism of the process, and its linear correlation indicates a diffusion-controlled mechanism.

The inset chart in Fig. 4A clearly shows a good linear relationship between I_p_ and ʋ^1/2^, indicating that the reaction is controlled by the diffusion process. Moreover, in the processes controlled by diffusion or adsorption, the slopes of the Log I_P_ vs. Log ʋ plot are 0.5 and 1, respectively. Figure 4B shows the logarithmic plot obtained from the change in the oxidation current of hydrazine at different potential scan rates, which has a slope of 0.4413 and confirms the control of the process with a diffusion process. Previous studies noted that in an irreversible reaction, there is a linear relationship between changes in peak potential (E_p_) and Log ʋ^5,54^.

The relevance of E_p_ and Log ʋ (Fig. 4C) shows a definite linear relationship with an acceptable coefficient of determination (R^2^ = 0.9913), which defines the irreversibility of the process. For this type of reaction, the following equations can be used for the determination of the electron transfer coefficient (α) (Eqs. 2 and 3) ^55^:

2 $${\text{E}}_{{\text{p}}} = \, \left( {{\text{b}}/{2}} \right){\text{ log }}\upsilon \, + {\text{ k}}$$

3 $$\alpha \, = { 1} - \, \left[ {{2}.{3}0{\text{3 RT}}/{\text{b n}}_{\alpha } {\text{F}}} \right]$$

In which b is double the slope in the E_p_ equation in Fig. 4C (E_p_ = 0.0805 log ʋ-0.3587), R, T, and F are 8.314 J mol^−1^ K^−1^, 298 K, and 96,500 C mol^−1^, respectively. n_α_ is the number of transferred electrons in the reaction rate-determining step, which is 1 in this process, and the overall calculations result in α = 0.6.

Considering the irreversible nature of hydrazine oxidation on Pd/PCz@GCE and the determined α = 0.6, the heterogeneous electron transfer rate constant (ks) was calculated using Laviron’s equation(Eq. 4) for irreversible systems^56^.

4 $${\text{Ep}} = {\text{E}}0 + \frac{{{\text{RT}}}}{{\alpha {\text{nF}}}}\left[ {\ln \left( {\frac{{{\text{RTks}}}}{{\alpha {\text{nF}}}}} \right) - \ln \upsilon } \right]$$

where n = 1, R = 8.314 J mol^−1^ K^−1^, T = 298 K, F = 96,500 C mol^−1^, ʋ is the potential scan rate in V s^−1^, E_p_ is the anodic peak potential, and E_0_ is the formal potential obtained through an iterative fitting procedure from E_p_–logʋ data (E_0_ = -0.4032 V vs. Ag/AgCl in this study). Fig. S4 shows the dependence of k_s_ on scan rate for the Pd/PCz@GCE electrode during hydrazine (1 mM) oxidation in 0.1 M NaOH.

The k_s_ values significantly increase as the scan rate rise from 0.01 to 0.5 V s⁻^1^, then levels off at higher scan rates. This pattern suggests that at low scan rates, increasing the potential sweep rate effectively speeds up the electron-transfer process, while at higher scan rates, inherent system limitations (such as diffusion or surface structural constraints) hinder further substantial improvements in electron-transfer kinetics. The maximum k_s_ value, approximately 6.9 s^−1^, occurs at intermediate to high scan rates, consistent with the reaction’s irreversible nature and diffusion-controlled mechanism. These findings demonstrate the high efficiency of Pd/PCz@GCE in facilitating electron transfer for hydrazine oxidation in alkaline media.

Alkaline media are preferred in electrochemical detection of hydrazine due to their ability to (1) stabilize the analyte^57^, (2) enhance oxidation kinetics, (3) suppress interfering signals^58^ and (4) allow detection at lower potentials^59^. These conditions collectively improve the analytical performance of the electrochemical sensor in terms of sensitivity, selectivity, and reproducibility. The main oxidation peak for palladium appears to have more positive potentials in acidic media than in alkaline media (on the RHE scale). Considering the selected potential range and the proposed possible mechanism, the role of OH^-^ as a reactant produces a complex that is the starting compound in the final oxidation process. The mechanism suggested for hydrazine oxidation in an alkaline medium is as follows (Eqs. 5, 6, 7)^55,60^:

5 $${\text{N}}_{{2}} {\text{H}}_{{4}} + {\text{ 2OH}}^{ - } \to \left[ {{\text{HO}}{-}{\text{H}}_{{2}} {\text{N}} = {\text{NH}}_{{2}} {-}{\text{OH}}} \right]^{{{2} - }}$$

6 $$\left[ {{\text{HO}}{-}{\text{H}}_{{2}} {\text{N}} = {\text{NH}}_{{2}} {-}{\text{OH}}} \right]^{{{2} - }} \to {\text{N}}_{{2}} {\text{H}}_{{2}} + {\text{ 2H}}_{{2}} {\text{O }} + {\text{ 2e}}^{ - }$$

7 $${\text{N}}_{{2}} {\text{H}}_{{2}} + {\text{ 2OH}}^{ - } \to {\text{N}}_{{2}} + {\text{ 2H}}_{{2}} {\text{O }} + {\text{ 2e}}^{ - }$$

$${\text{N}}_{{2}} {\text{H}}_{{4}} + {\text{ 4HO}}^{ - } \to {\text{N}}_{{2}} + {\text{ 4H}}_{{2}} {\text{O }} + {\text{ 4e}}^{ - } \left( {\text{overall reaction}} \right)$$

Pd/PCz@GCE can catalyze the oxidation of hydrazine by facilitating electron transfer at the rate-determining step, resulting in the occurrence of an electrochemical reaction at lower potentials with higher current intensity. The catalytic effect may be attributed to the synergistic effects of the carbazolic conductive polymer and Pd nanoparticles, which are distributed on the PCz to improve the conductivity and kinetics of electron transfer. This distinct synergistic interaction, facilitated by the uniform distribution of Pd nanoparticles within the innovative polycarbazolic matrix, is a crucial factor contributing to the outstanding electrocatalytic activity and sensitivity of the sensor, thus representing a significant advancement in electrochemical hydrazine detection.

#### Amperometric determination

**Fig. 5:**
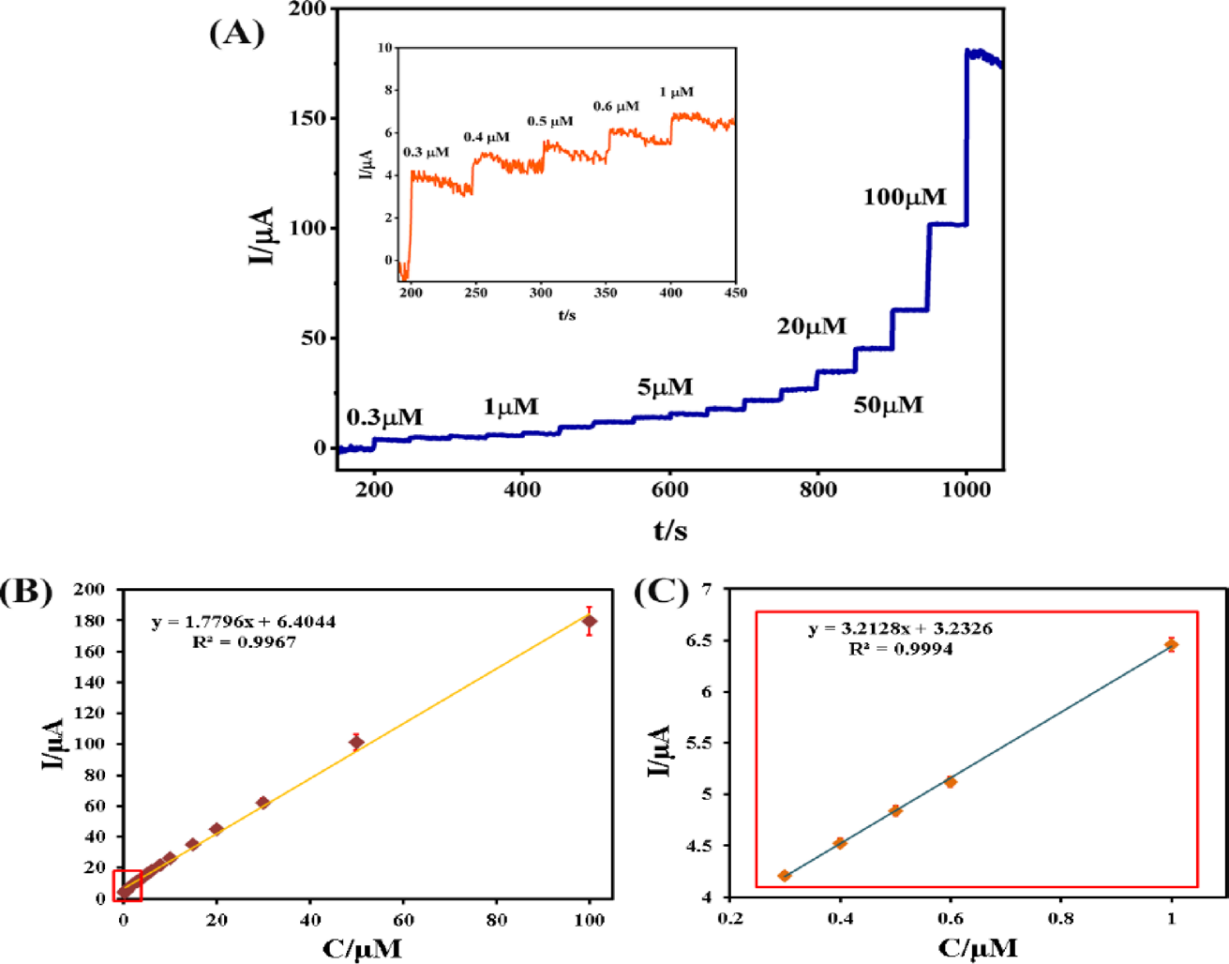
(**A**) Amperometric response of Pd/PCz@GCE during the successive addition of hydrazine at concentrations ranging from 0.3 to 100 μM, including responses at 0.3–1 μM (inset) in 0.1 M NaOH solution. Current vs. hydrazine concentration plot in the range of (**B**) 0.3 to 100 μM and (**C**) 0.3–1 μM.

The amperometric measurements were conducted to assess the electrocatalytic properties of the Pd/PCz@GCE at an applied potential of − 0.41 V vs. Ag/AgCl or in a continuously stirred basic medium (0.1 M NaOH solution). As shown in Fig. 5A, the amperogram of Pd/PCz@GCE exhibited a sensitive response to the addition of hydrazine at concentrations ranging from 0.3 to 100 μM, in which the current intensity of the oxidation peak reached an acceptable steady state within a few seconds, indicating the electrocatalytic ability of Pd/PCz@GCE for the oxidation of hydrazine. For more clarity, the inset image in Fig. 5A depicts the amperometric current responses for low concentrations of hydrazine (0.3–1 μM).

Figure 5B shows the plot of current (μA) vs. hydrazine concentration (μM) for Pd/PCz@GCE, which displays a linear relationship in the range from 0.3 to 100 μM with a determination coefficient (R^2^) of 0.997. Figure 5C shows the extended plot of current vs. hydrazine concentration for the low-concentration region, which is specified by the red square in Fig. 5B. On the basis of the line equation (y = 1.7796x + 6.4044) and the definition of sensitivity^61^ as the division of slope to the electrode surface (2 mm diameter), the sensitivity of Pd/PCz@GCE reached 56.64 μA μM^−1^ cm^−2^. The calculated limit of detection (LOD) was 3.3 times the standard deviation of the blank, and the slope of the plot was 0.084 μM. Table 1 shows a comparison of the electroanalytical efficiency of Pd/PCz@GCE with that of several other reported electrochemical sensors for hydrazine determination. The results show that the sensitivity, LOD, and linear range of the Pd/PCz@GCE are comparable and even better than those of the other proposed electrodes. The significant efficiency of Pd/PCz@GCE for the determination of hydrazine can be attributed to the presence of well-distributed Pd nanoparticles in the conductive polycarbazolic polymer on the surface of the electrode, which facilitates and accelerates the oxidation of hydrazine.

**Table 1:** Comparison of electroanalytical efficiency of some reported sensor for hydrazine determination.

Electrode	Medium	Sensitivity (μA μM^−1^ cm^−2^)	Linear range (μM)	LOD (μM)	Ref
PEDOT/MWCNT/Pd/GCE	Phosphate buffer, pH = 6.86	40	0.5–5000	0.8	^62^
Pd NPs–PANI/GCE	Phosphate buffer, pH = 7	0.5	10–300	0.06	^63^
Pd-MWCNTs/GCE	0.5 M H_2_SO_4_	0.146	0.1–10	0.016	^64^
Pt–Pd/ERGO/GCE	0.1 M NaOH , pH = 13	8	7–5500	1.7	^65^
Pd PEDOT@CM/GNP/GCE	Phosphate buffer, pH = 7	0.2	1–5000	0.3	^66^
Pd NPs-EGNS/SPCE	0.1 M KCl solution, pH = 7	4.4	0.05–1415	0.004	^67^
Se/PGE	0.1 M K₂SO₄, pH = 7	0.813	1 – 50,000	0.045	^68^
Pd/PCz@GCE	0.1 M NaOH, pH = 13	**56.64**	**0.3–100**	**0.084**	**This work**

PEDOT/MWCNT/Pd: poly-3,4-ethylenedioxythiophene/multi-wall carbon nanotube/palladium; Pd NPs–PANI: Palladium Nanoparticles- Polyaniline; Pd-MWCNTs: Palladium- multi-wall carbon nanotubes; Pt–Pd/ERGO: Platinum-Palladium/electrochemically reduced graphene oxide; Pd-PEDOT@CM/GNP/GCE: Palladium- poly-3,4-ethylenedioxythiophene@ carbon microspheres/graphene nanoplatelets; Se/PGE :Selenium nanostructure-coated Pencil Graphite Electrode via CBD

Significant values are in [bold]

**Fig. 6:**
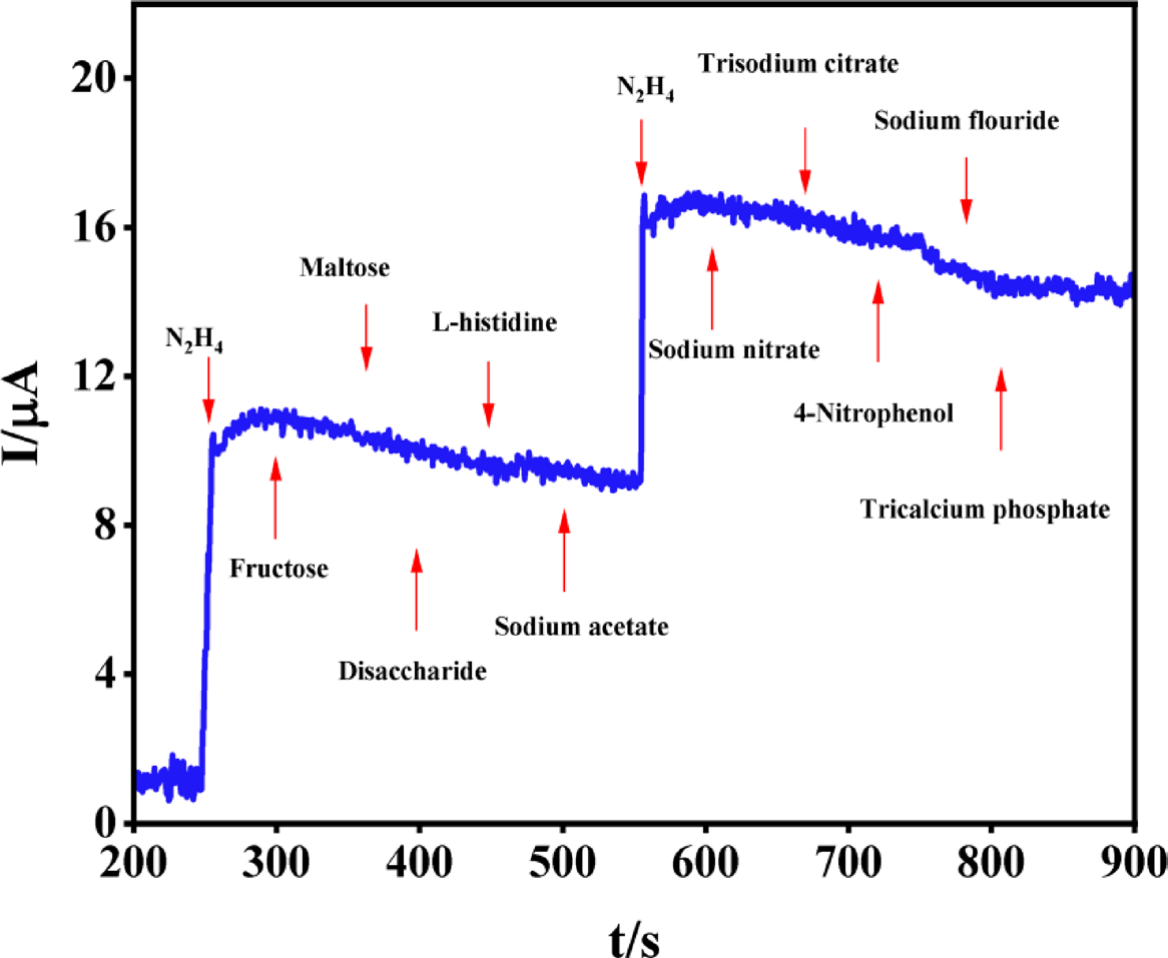
Amperometric response of Pd/PCz@GCE to injection of hydrazine at concentrations of 2 μM for the first step and 5 μM for the second step in the presence of 20-fold excess (100 μM) concentrations of fructose, maltose, disaccharide, L-histidine, sodium acetate, sodium nitrate, trisodium citrate, 4-nitrophenol, sodium fluoride and tricalcium phosphate in 0.1 M NaOH solution.

The effects of different compounds, including fructose, maltose, disaccharides, L-histidine, sodium acetate, sodium nitrate, trisodium citrate, 4-nitrophenol, sodium fluoride, and tricalcium phosphate, on the oxidation current were examined to evaluate the selectivity of the Pd/PCz@GCE. Figure 6 shows the fast amperometric response of Pd/PCz@GCE to the addition of 2 and 5 μM hydrazine (first and second steps, respectively) in the basic medium (0.1 M NaOH solution), whereas the current response did not significantly change during the addition of the interferents at a 20-fold excess (100 μM) concentration. This result reveals the excellent selectivity of Pd/PCz@GCE for the determination of hydrazine.

#### Reproducibility, stability and repeatability

The accuracy and reproducibility of the Pd/PCz@GCE^69^ were evaluated in response to 1 mM hydrazine in 0.1 M NaOH solution and for six different electrodes. As shown in Fig. S5, the results clearly define the reproducible process of electrode fabrication, confirmed by the reliable relative standard deviation (RSD) of 0.69%. The accurate performance of the electrode is evident from the peak currents (inset A) and oxidation potential (inset B) for all six measurements.

The repeatability and stability of the Pd/PCz@GCE were also investigated to confirm its reliable performance^5,69^. Fig. S6 shows the current density resulting from the oxidation process of 1 mM hydrazine, which was performed by the Pd/PCz@GCE in five consecutive separate runs, including cleaning and washing of the sensor at the end of each run. The Pd/PCz@GCE clearly has excellent repeatability, with an RSD of 0.68%. The stability of the Pd/PCz@GCE was evaluated by testing the consistency of the current density in the oxidation process of 1 mM hydrazine in 0.1 M NaOH solution at a constant potential of -0.41 V for long-term use. Fig. S6 reveals the excellent stability of the Pd/PCz@GCE, as there was no significant change in current density during the oxidation of the hydrazine-containing sample even after 7000 s, and only a slight decrease of approximately 3% in current density occurred after 12,000 s. The results indicate that the Pd/PCz@GCE has excellent reproducibility, repeatability and stability for the electrochemical detection and determination of hydrazine.

#### EIS

EIS was used to evaluate the limitations of the reaction kinetics on the surface of the Pd/PCz@GCE. The Nyquist plots of the GCE, PCz/GCE, and Pd/PCz@GCE samples were recorded at a 1:1 (1.0 mM) molar ratio of K_3_Fe(CN)_6_/K_4_Fe(CN)_6_ and 0.1 M KCl with the application of a 10-mV perturbation amplitude at 100 kHz to 0.01 Hz sweeping frequency (Fig. S8). The Nyquist plot is composed of a semicircle and a straight line in the higher and lower frequency ranges, respectively. The linear section has a slope lower than 90°, which defines the capacitive behaviour of the conductive polymer. The resistance to charge transfer manifests in the diameter of the semicircle^70^. As seen between the observable sections of the semicircles at high frequency, the semicircle corresponding to Pd/PCz@GC has the smallest diameter, indicating less charge transfer resistance (R_ct_) and the least limitation in electron transfer between the electrolyte and electrode. The conductive polymer film on the surface of the electrode has a resistance that can be ascribed to charge accumulation in the surface proximity of the polymer film and the interface of the electrolyte^49^. The decrease in R_ct_ may be ascribed to the conductivity of the carbazolic polymer and the activity of the embedded Pd nanoparticles, which can facilitate the kinetics of charge transfer.

#### Determination of hydrazine in real samples

Table 2 shows the determination of hydrazine in mineral water, river water, and tap water samples via the Pd/PCz@GCE electrochemical sensor. Hydrazine was spiked at concentrations of 20, 40, and 70 μM, and the found concentrations, recovery percentages, and relative standard deviations (RSD, n = 3) were calculated to assess method performance. The results underscore the exceptional precision and reproducibility of the method across diverse aqueous matrices. In mineral water, recoveries ranged from 98.90 to 99.93%, with RSD values between 0.63 and 3.98%, demonstrating high accuracy and excellent reproducibility, particularly at relatively low concentrations (RSD of 0.63% at 20 μM). For river water, recoveries were slightly elevated at 100.60 to 101.30%, with RSDs of 3.54 to 4.01%, reflecting robust accuracy and acceptable reproducibility (RSD < 5%)^71^ despite the complex matrix. In tap water, the recoveries ranged from 98.69 to 99.10%, with RSDs ranging from 0.97 to 3.46%, indicating strong performance even in the presence of potential interferents such as residual chlorine. Overall, the method achieved recoveries within the standard analytical range of 95 to 105% (98.69 to 101.30%) and RSDs of 0.63 to 4.01%, highlighting its superior precision and reproducibility. These attributes position the method as highly suitable for the environmental monitoring of hydrazine, particularly in cleaner matrices such as mineral water, where it exhibits optimal performance.

**Table 2:** Determination of hydrazine in mineral, river, and tap water samples using Pd/PCz@GCE electrochemical sensor.

Sample	Hydrazine added (μM)	Hydrazine found (μM)	Recovery (%, n = 3)	RSD (%)
Mineral water	20.00	19.78	98.90	0.63
	40.00	39.86	99.65	2.73
	70.00	69.95	99.93	3.98
River water	20.00	20.12	100.60	3.93
	40.00	40.43	101.08	4.01
	70.00	70.91	101.30	3.54
Tap water	20.00	19.82	99.10	0.97
	40.00	39.56	98.90	2.29
	70.00	69.08	98.69	3.46

Additionally, the performance of the Pd/PCz@GCE sensor was evaluated against established methods for hydrazine detection, including HPLC with various detectors and commonly used fluorescent probes. The comparative results, which summarize detection limits, linear ranges, sensitivities, and practical considerations, are provided in Table S1.

#### Electrochemical surface area (ECSA)

The electrochemically active surface area (ECSA) of the Pd/PCz@GCE electrode was determined using the PdO-reduction method^72^.

CV curves were recorded within the potential range –0.22 V to –0.67 V at a scan rate of 50 mV s⁻^1^ (Fig. S9). The PdO-reduction charge (Q) was calculated by integrating the cathodic area associated with Eqs. (8):

8 $${\text{PdO}} + {\text{2H}}^{ + } + {\text{2e}}^{ - } \to {\text{Pd}} + {\text{H}}_{{2}} {\text{O}}$$

Following the conversion of current density to total current, the integration yielded Q = 964.92 μc. Using the literature value of S = 405 µC cm^−2^ as the characteristic reduction charge density for a monolayer of PdO to Pd^73^, the ECSA was calculated using Eq. (9):

9 $${\text{ECSA }} = {\text{ Q}}/{\text{S}}$$

The ECSA is measured to be 2.38 cm^2^, showing that the electroactive surface is roughly 76 times larger than the geometric area of the bare GCE. This significant increase confirms the high surface roughness and abundance of catalytically active Pd sites introduced by the PCz matrix and Pd nanoparticle modification, which is expected to greatly enhance the electrocatalytic performance of electrode in hydrazine oxidation.

### Conclusions

In summary, a novel electrochemical sensor (Pd/PCz@GCE) has been successfully developed for the highly sensitive and selective determination of hydrazine. The electrochemically deposited polycarbazolic conductive polymer served as an effective substrate for the electroreductive embedding of Pd nanoparticles, resulting in a sensor with remarkable electrocatalytic activity toward hydrazine oxidation in a basic medium. The Pd/PCz@GCE sensor exhibited a wide linear range (0.3–100 μM). The Pd/PCz@GCE sensor exhibited a wide linear range (0.3–100 μM), a low LOD (0.084 μM), and high sensitivity (56.64 μA μM^−1^ cm^−2^). Furthermore, the sensor demonstrated excellent selectivity against common interferents, as well as remarkable reproducibility, repeatability, and stability. Kinetic studies revealed a diffusion-controlled mechanism at the electrode surface. Compared with other reported electrochemical sensors (Table 1), the Pd/PCz@GCE sensor exhibits comparable or superior performance in terms of sensitivity, LOD, and linear range, which is attributed to the synergistic effect of the well-dispersed Pd nanoparticles within the conductive polycarbazolic polymer matrix. The practical applicability of the sensor was validated by determining hydrazine in mineral water, river water, and tap water samples (Table 2). The results revealed excellent recoveries (98.69% to 101.30%) and RSD values (0.63% to 4.01%), highlighting the method’s precision and reproducibility in diverse aqueous matrices. This study successfully demonstrates a new approach for highly sensitive hydrazine detection by creating a unique polycarbazolic polymer-palladium nanoparticle composite, which marks a significant advancement for the electrochemical determination of hydrazine in real-world applications, especially for environmental use monitoring.

## Experimental

### Materials, methods and instruments

All chemicals, including carbazole (95%), ammonium persulfate (APS, 98%), palladium/charcoal activated (Pd/C-10% Pd), 4-fluoronitrobenzene (99%), anhydrous potassium carbonate (K_2_CO_3_, 99%), hydrazine hydrate (80%), ethanol (EtOH, 99.8%), N,N-dimethylformamide (DMF, 99.8%), hydrochloric acid (HCl, 37%), dimethyl sulfoxide (DMSO, 99.8%), tetrabutylammonium tetrafluoroborate (TBABF_4_, 99%), palladium chloride (PdCl_2_, 99%), fructose, maltose monohydrate, L-histidine (99%), sodium acetate trihydrate (99%), sodium nitrate (99%), trisodium citrate dehydrate (99%), 4-nitrophenol (99%), sodium fluoride (99%), and alpha-tricalcium phosphate (98%), were purchased from Merck and used without any purification. Cyclic voltammetry (CV) and amperometry were carried out via an Autolab 302 N galvanostat/potentiostat (Netherlands). A three-electrode cell consisting of a working electrode (modified glassy carbon electrode, 3.0 mm diameter), an auxiliary electrode (platinum-wire electrode), and a reference electrode (Ag|AgCl, 3 M KCl) was employed. Electrochemical impedance spectroscopy (EIS) was performed at the open circuit potential (OCP) in a cell containing a 1:1 (1.0 mM) molar ratio of K_3_Fe(CN)_6_/K_4_Fe(CN)_6_ and 0.1 M KCl. A 10 mV perturbation amplitude and 100 kHz to 0.01 Hz sweeping frequency were applied. In addition, to remove oxygen from the solution during each test, N_2_ gas was added to the solutions. Fourier transform infrared (FT-IR) spectra were recorded on a Tensor 27 FT-IR Bruker spectrometer (Germany) in the KBr matrix. Field emission scanning electron microscopy (FE-SEM, TESCAN MIRA3) and energy dispersive X-ray spectroscopy (EDX) were used to investigate the surface morphology and perform elemental mapping analysis. A Perkin Elmer STA 6000 series instrument was used for thermal analysis. Thermogravimetric analysis (TGA) was performed at a temperature range of 50–600 °C and a scanning rate of 10 °C/min under a N_2_ atmosphere. Differential scanning calorimetry (DSC) was carried out at the same temperature range by applying a heating rate of 10 °C/min under a N_2_ atmosphere. Powder X-ray diffraction data were collected on a Philips PW1730 powder diffractometer using Cu K_α_ radiation operating at 40 kV and 30 mA at 293 K.

### Synthesis of the carbazole Monomer

#### Synthesis of the precursor carbazole: *N*-(4-nitrophenyl)carbazole

The same mole amounts (60 mmol) of carbazole (10.02 g), 4-fluoronitrobenzene (9.30 g), and potassium carbonate (9.06 g) were added to a 150 mL round-bottom flask containing 75 mL of DMF and stirred for 10 min under a N_2_ atmosphere at room temperature. The mixture was then heated to reach a reflux state at 160 °C and stirred under N_2_ flux for 24 h. After the reaction was complete, heating was stopped, and the mixture was cooled to room temperature, followed by slow pouring into ice water. A yellowish flocculent solid was obtained during slow stirring of this mixture, and after complete melting on ice, the resulting precipitate was separated and dried by suction filtration. The mixture was then washed successively with water (8 times), distilled water (4 times), and anhydrous methanol (4 times) followed by drying at 60 °C in a vacuum oven for 12 h, yielding 16.60 g of yellowish powder with a 96% yield of N-(4-nitrophenyl)carbazole (Fig. S8).

#### Synthesis of carbazolic monomer: *N*-(4-aminophenyl)carbazole

Certain amounts of monomer precursor (4.30 g, 15 mmol) and hydrogenation catalyst (0.35 g, 10% Pd/C) were sequentially added to a 150 mL three-necked flask, followed by the addition of ethanol (25 mL). The mixture was stirred well and then heated to 60 °C. Then, hydrazine hydrate (13 mL) was added to the reaction mixture through a constant pressure dropping funnel within almost one hour at a rate of 3–4 drops per minute, and the mixture was stirred for 12 h at approximately 60 °C. After the completion of the reaction, the hydrogenation catalyst was separated by filtration through a sand core funnel, and ethanol and hydrazine hydrate were distilled from the filtrate at a reduced temperature. A white precipitate was obtained after the residual liquid was poured into the water, which was subsequently washed 5 times with distilled water and dried at 60 °C for 12 h in a vacuum oven. The product was recrystallized from absolute ethanol at 85 °C under a N_2_ atmosphere, and the resulting crystals were dried in a vacuum oven at 60 °C for 12 h to yield 2.77 g brown crystals of *N*-(4-aminophenyl) carbazole as a monomer carbazole in 71.4% yield from the monomer precursor (Fig. S8).

### Polymer synthesis: Poly(N-(4-aminophenyl)carbazole)

A total of 0.5 g of carbazolic monomer was added to a 50 mL two-necked round-bottom flask containing 5 mL of DMSO, and the mixture was stirred at room temperature until completely dissolved. After 30 min, a solution of APS (2 g) in 2 mL of HCl (1 M) was added to the solution under N_2_ flux, and the mixture was stirred overnight at ambient temperature. The resulting mixture was then poured into 200 mL of a washing solution containing a water/methanol mixture (7:3 v/v) and stirred. The precipitate was separated by centrifugation and purified through a 50–50 water‒methanol mixture via a Soxhlet extractor within 24 h. The polymer was then collected and dried in a vacuum oven at 40 °C for 24 h at 70% yield as a black powder. The synthetic route of the polymer is shown in Fig. S8.

### Electrochemical preparations

#### Preparation of the polymer-modified GCE

The GCE was polished with alumina powder (0.05 μm) for 5 min and then washed with distilled water, followed by sonication in a mixture of water and ethanol (1:1, V: V) for 5 min to remove the remaining alumina particles. The modified electrode was prepared via the polymerization of a film from a mixture containing dispersed synthesized carbazolic polymer (0.002 g) in a solution of TBABF_4_ (0.342 g) in 10 mL of DMSO as a supporting electrolyte at the surface of the working GCE via CV. The potential range was − 2 to 1 V at a scanning rate of 50 mV s^−1^ for 3 cycles. The electrode was then rinsed with DMSO to remove any unbound material from the surface and dried at 50 °C for 1 h to form the PCz@GCE.

#### Pd promotion of the polymer-modified GCE

Preparation of the final palladium-modified electrode was carried out in two steps. In the first step, the prepared electrode, PCz@GCE, was immersed in an acidic 5 mM solution of PdCl_2_ as the source of embedded Pd^2+^ cations for 15 min and then dried at room temperature. In the second step, the CV technique was used for the reduction of embedded Pd^2+^ cations in PCz@GCE at a potential window of 0.25 to 1.2 V and a scan rate of 50 mV s^−1^ for 3 cycles in 0.5 M H_2_SO_4_ solution. The sample was then rinsed with deionized water and denoted as Pd/PCz@GCE.

## Supplementary Information

Below is the link to the electronic supplementary material.

Supplementary Material 1

## Data Availability

All data generated or analysed during this study are included in this published article.
