# Supplementary material for: Novel conductive polycarbazolic polymer embedded with palladium nanoparticles as a highly sensitive electrochemical sensor for hydrazine detection
Source: Sci Rep. 2025 Nov 26;15:42199. doi: 10.1038/s41598-025-25980-8 (PMC12657885; doi:10.1038/s41598-025-25980-8)
Supplement: Supplementary file 1 — Supplementary Material 1 [file 41598_2025_25980_MOESM1_ESM.docx]

**Supplementary information**

**Novel Conductive Polycarbazolic Polymer Embedded with Palladium Nanoparticles as a Highly Sensitive Electrochemical Sensor for Hydrazine Detection**

*Moghadaseh Aghaei Araei^1^, Moslem Mansour Lakouraj^1*^, Shahram Ghasemi^2^, Rahman Hosseinzadeh^1^*

*^1^Department of Organic-Polymer Chemistry, Faculty of Chemistry, University of Mazandaran,*

*Babolsar, Iran*

*^2^Department of Nano-Chemistry, Faculty of Chemistry, University of Mazandaran,*

*Babolsar, Iran*

** Corresponding author:* [*lakouraj@umz.ac.ir*](mailto:lakouraj@umz.ac.ir)

Fig.S1. Considered structure of Poly(N-(4-aminophenyl)carbazole)


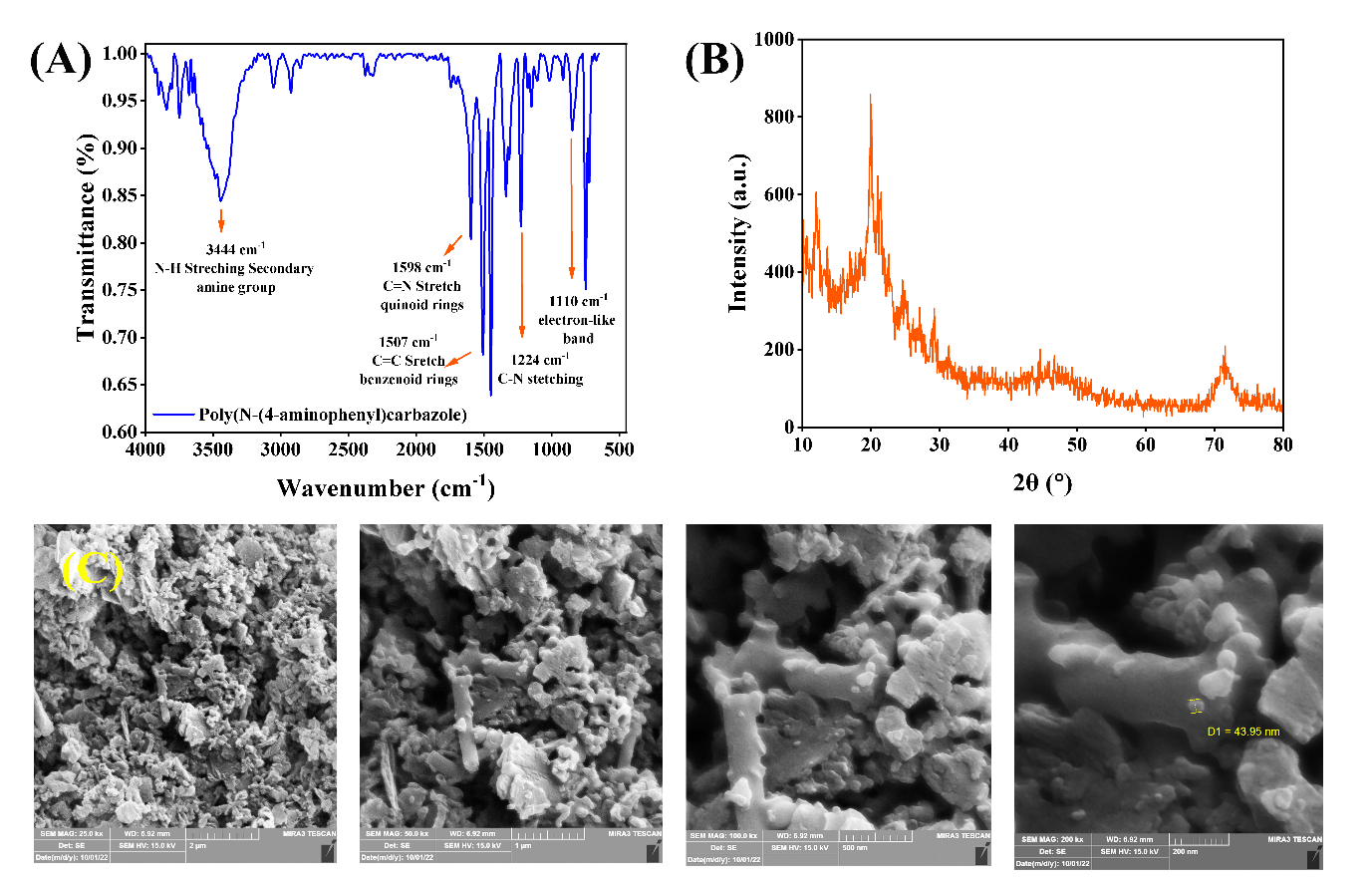


Fig. S2: (A) FT-IR spectra, (B) XRD and (C) FESEM images (at different magnifications) of polymer.

Fig. S3: (A) TGA and DSC thermograms and (B) UV-Vis spectrum of monomer and polymer.

Fig. S4: Dependence of the heterogeneous electron-transfer rate constant (ks) on scan rate for the Pd/PCz@GCE electrode during hydrazine (1 mM) oxidation in 0.1 M NaOH.

Fig S5: Reproducibility of the Pd/PCz@GCE as the responses of six different electrodes to oxidation of 1 mM hydrazine in 0.1 M, NaOH solution and at scan rate of 0.5 V s^-1^, showing performance accuracy in A) peak currents and B) oxidation potential.

Fig S6: Current density changes during oxidation process of 1 mM hydrazine in basic medium (0.1 M, NaOH solution) by the Pd/PCz@GCE as A) repeatability at five consecutive separate run (including cleaning and washing of sensor at the end of each run) and B) stability at a constant potential of -0.41 V for a long term run.


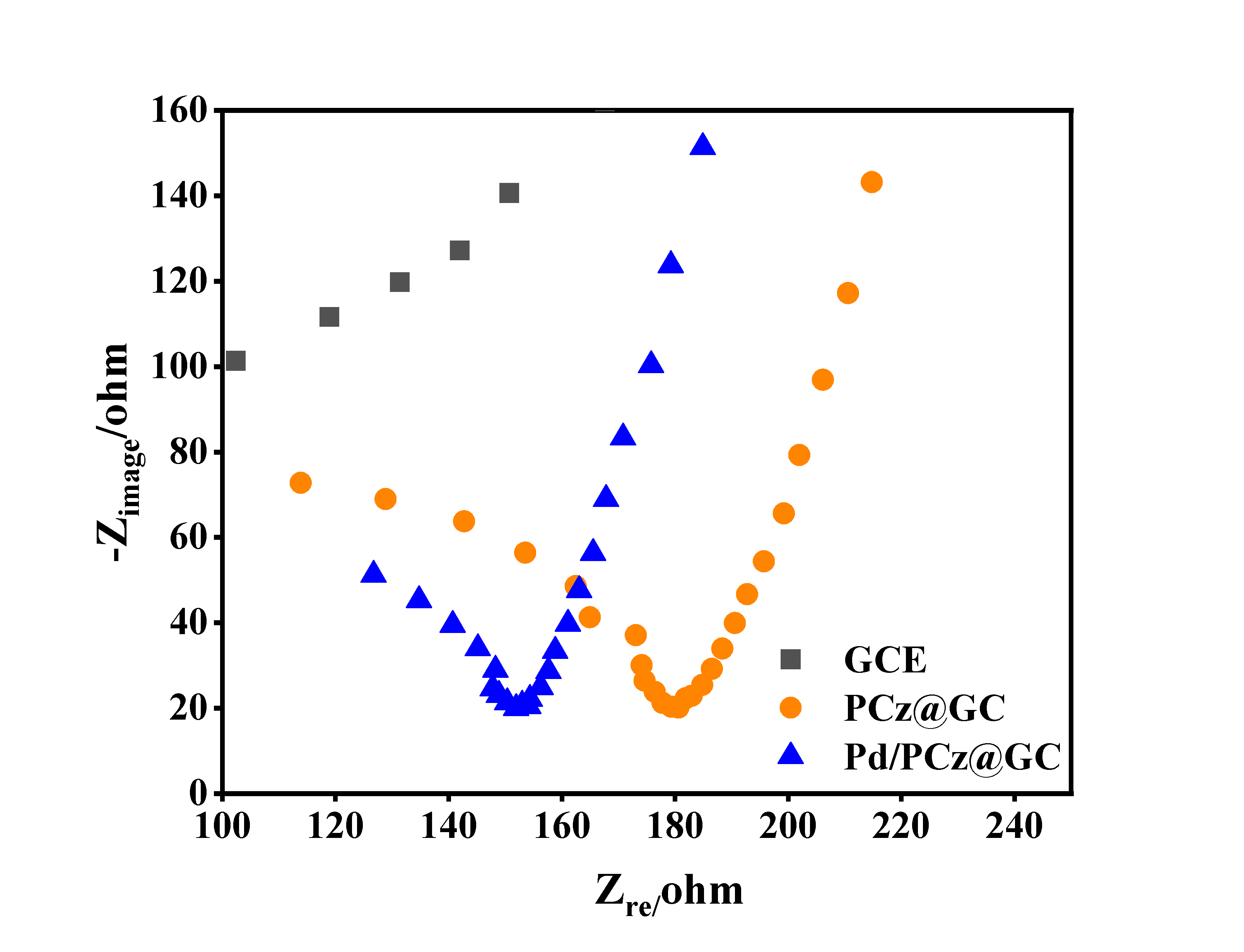


Fig. S7: Nyquist plot of GCE, PCz/GCE and Pd/PCz@GCE at OCP in 1.0 mM K_3_Fe(CN)_6_/K_4_Fe(CN)_6_ and 0.1 M KCl

Fig.S8. Monomer and polymer synthesis procedure

Fig.S9. Cyclic voltammogram showing the procedure for determining the electrochemical surface area (ECSA) of the Pd/PCz@GCE electrode in 1 M NaOH at a scan rate of 50 mV s⁻¹ at ambient temperature.

**Table S.1: Comparative Analysis of Hydrazine Detection Methods and the Developed Pd/PCz@GCE Sensor**

| Method / Sensor | LOD (µM) | Linear Range (µM) | Sensitivity (µA·µM⁻¹·cm⁻²) | Sample Prep. | Cost / Complexity | Ref |
| --- | --- | --- | --- | --- | --- | --- |
| **Pd/PCz@GCE (this work)** | **0.084** | **0.3–100** | **56.64** | **Simple** | **Low** | **This work** |
| Fluorescent probe 1 (Rapid detection of hydrazine…) | 0.11 | 0–100 | - | Simple | Low–Med | [1] |
| Fluorescent probe (2 fluoro 5 nitro benzoic ester masked) | 0.10 | 0–20 | - | Simple | Low | [2] |
| HPLC–MS–MS (p‑anisaldehyde deriv., isotope dilution) | 0.0015 | 0.0015–0.384 | - | Complex | High | [3] |
| HPLC–MS/MS (p‑tolualdehyde deriv.) | 0.0000625 | 0.000156–1.56 | - | Complex | High | [4] |
| HPLC–UV–MS/MS (2‑quinolinecarboxaldehyde, UDMH) | 0.0000615–0.00216 | 0.000166–16.64 | - | Complex | Med–High | [5] |
| UV–Vis ES PANI (AOT‑doped) | - | - | - | Simple | Low–Med | [6] |

Table S.1 provides a detailed comparison between the developed Pd/PCz@GCE electrochemical sensor and established methods for hydrazine detection, including High-Performance Liquid Chromatography (HPLC) with various detectors (UV–Vis, MS/MS, fluorescence) as well as commonly used fluorescent probes. While some HPLC–MS/MS techniques achieve very low detection limits, they require multi-step derivatisation, specialised and costly equipment, and lengthy analysis times. In contrast, the Pd/PCz@GCE, with a sensitivity of 56.64 µA_·_µM⁻¹_·_cm⁻², a detection limit of 0.084 µM, and a linear range of 0.3–100 µM, offers straightforward sample preparation, a quick response, and minimal equipment requirements. Its design —a polycarbazole film decorated with palladium nanoparticles on a glassy carbon electrode— is easily adaptable for portable and online monitoring systems. This combination of analytical performance, ease of operation, and low cost makes it ideal for real-time hydrazine monitoring in industrial, environmental, and biological settings, as demonstrated in Section 2.2.8 for real-world samples, including mineral water, tap water, and industrial wastewater.

Refrencess

[1] Jung, M. J., Kim S. J.& Lee M. H. π-Extended tetraphenylethylene containing a dicyanovinyl group as an ideal fluorescence turn-on and naked-eye color change probe for hydrazine detection. *ACS omega*. **5,** 28369-74 (2020).

[2] Zhai, Q., Feng W.& Feng G. Rapid detection of hydrazine in almost wholly water solution and in living cells with a new colorimetric and fluorescent turn-on probe. *Analytical Methods*. **8,** 5832-7 (2016).

[3] Isenberg, S. L., Carter M. D., Crow B. S., Graham L. A., Johnson D., Beninato N., Steele K., Thomas J. D.& Johnson R. C. Quantification of Hydrazine in Human Urine by HPLC–MS-MS. *J. Anal. Toxicol.* . **40,** 248-54 (2016).

[4] Song, L., Gao D., Li S., Wang Y., Liu H.& Jiang Y. Simultaneous quantitation of hydrazine and acetylhydrazine in human plasma by high performance liquid chromatography-tandem mass spectrometry after derivatization with p-tolualdehyde. *J. Chromatogr. B*. **1063,** 189-95 (2017).

[5] Timchenko, Y. V., Stavrianidi A. N., Smolenkov A. D., Pirogov A. V.& Shpigun O. A. A novel simple and sensitive approach for determination of 1, 1-dimethylhydrazine in aqueous samples by high performance liquid chromatography with ultraviolet and tandem mass spectrometric detection after derivatization with unsubstituted aromatic aldehydes. *Chemosphere*. **280,** 130747 (2021).

[6] Sabri, F. N., Monajemi H., Zain S. M., Wai P. S., Rungrotmongkol T.& Lee V. S. Molecular conformation and UV–visible absorption spectrum of emeraldine salt polyaniline as a hydrazine sensor. *Integr. Ferroelectr.* **175,** 202-10 (2016).
